# Four Letters with Replies

**Published:** 1876-01

**Authors:** 


### Our Correspondence.

BARNESVILLE, Pike Co., Ga., Oct. 14th, 1875.

THAD S. UP DE GRAFF, M. D.:

*Dear Doctor.*—Your article on Cataract in the October No. of the BISTOURY, is charmingly interesting, and I write to suggest, modestly, if it would not be a blessing to us younger members of the profession, if, in each number, you would give this much space tò some of the diseases of the eye, which are so little understood by the profession at large. Very Respectfully,

C. S. S., M. D.

Thanks for the appreciation expressed. We continue the articles, as you preceive, in this number.

---

OSWEGO, N. Y., Dec. 12, 1875.

DR. UP DE GRAFF:

Enclosed subscription price of BISTOURY for another year. The article on examination of urinary deposits, is alone worth many times what you ask for the journal. I never before so fully received the worth of my money, in any periodical, as I do in the BISTOURY. I enclose money for a new subscriber, to whom send as indicated below.

J. R. F.

We receive many congratulatory letters, like the above, from subscribers in all parts of the United States. Of course, we are delighted to know that the BISTOURY is appreciated. Thanks for your kind words.

---

POUGHKEEPSIE, N. Y., Nov. 20, 1875.

EDITOR BISTOURY:

We have a little child, two years old, who is cross-eyed. Became so after a violent whooping-cough. We desire to have her eyes straightened, and write you to learn at what age it should be done. You can reply through the BISTOURY for the information of others who have asked us the question.

B. R.

In cross-eye, after the child is old enough to use the eyes in looking at small objects, for amusement or employment, or in reading—say from four to eight years of age, they acquire the habit of disusing one eye—the crooked one, entirely; that is, they suppress the image formed in the deviating eye, in order to prevent their seeing double. If this habit is continued for several years, the squinting eye is permanently disabled, often becoming blind. An operation therefore should be made as early as possible after the ages indicated.

---

SMITHVILLE, Lawrence Co., Ark., Oct. 3d., 1875.

DR. THAD S. UP DE GRAFF:

*Dear Sir.*—I drop you a few lines in behalf of my eyes. I am 45 years of age, eyes black, have been very strong sighted. My occupation is à miller and mill-wright, which is hard on the eyes. I have lived in the western winds a few years, and have had frequently small bits of steel in my eyes from the pick, while dressing mill-burrs. My eyes are fast failing me, and I wish to know what you would advise for me, and whether you could furnish me with a pair of spectacles that would suit my eyes. I am of dark complexion, dark hair and eyes, and have generally been healthy. Please send me your advice and price of glasses mounted in gold and silver. State how to send money and much oblige, D. R.

Here is a letter from an honest, hard-working man, anxious for relief for his dim eyes, but utterly ignorant of the manner in which glasses should be fitted. We could no more send him glasses, adapted to his eyes, and that would be safe for him to wear, without first testing his eyes, in order to learn what the difficulty to perfect vision is, than we could fit a garment to his body by his indicating when he cut his eye teeth.

We are constantly brought in contact with persons whose eyes have been seriously injured by the use of improper spectacles. People are in the habit, when their eyes begin to fail, to purchase of the nearest jeweler, or the first spectacle vender that enters their door, glasses of their own selection or fancy, glasses that magnify print sufficiently to enable them to read, but usually of too high power, the use of which soon enfeebles the power of accommodation. The proper way to adapt glasses to your eye is first to consult an oculist, who, with test glasses, will fit you accurately. When this cannot be done, you must rely upon your own judgment, which can be aided in this manner:—Go to your spectacle dealer, sit with your back to the light, take a tray of glasses in your lap, and try on various powers, until you find a pair that will meet the following requirements: With the newspaper 15 inches from your face, the glasses that will render the print distinct, without magnifying it, and without producing a bright and colored halo about the letters, and which, after a half hour or hour's use, will not leave the eyes feeling fatigued, will come as near fitting you as can be accomplished by your own judgment.

